# Exploring the GDB-13 chemical space using deep generative models

**DOI:** 10.1186/s13321-019-0341-z

**Published:** 2019-03-12

**Authors:** Josep Arús-Pous, Thomas Blaschke, Silas Ulander, Jean-Louis Reymond, Hongming Chen, Ola Engkvist

**Affiliations:** 1 Hit Discovery, Discovery Sciences, IMED Biotech Unit, AstraZeneca, Gothenburg, Pepparedsleden 1, 43183 Mölndal, Sweden; 2 Medicinal Chemistry, Cardiovascular, Renal and Metabolism, IMED Biotech Unit, AstraZeneca, Gothenburg, Pepparedsleden 1, 43183 Mölndal, Sweden; 3 Department of Chemistry and Biochemistry, University of Bern, Freiestrasse 3, 3012 Bern, Switzerland; 4 Department of Life Science Informatics, B-IT, LIMES Program Unit Chemical Biology and Medicinal Chemistry, Rheinische Friedrich-Wilhelms-Universität, Endenicher Allee 19C, 53115 Bonn, Germany

**Keywords:** Deep learning, Chemical space exploration, Deep generative models, Recurrent neural networks, Chemical databases

## Abstract

Recent applications of recurrent neural networks (RNN) enable training models that sample the chemical space. In this study we train RNN with molecular string representations (SMILES) with a subset of the enumerated database GDB-13 (975 million molecules). We show that a model trained with 1 million structures (0.1% of the database) reproduces 68.9% of the entire database after training, when sampling 2 billion molecules. We also developed a method to assess the quality of the training process using negative log-likelihood plots. Furthermore, we use a mathematical model based on the “coupon collector problem” that compares the trained model to an upper bound and thus we are able to quantify how much it has learned. We also suggest that this method can be used as a tool to benchmark the learning capabilities of any molecular generative model architecture. Additionally, an analysis of the generated chemical space was performed, which shows that, mostly due to the syntax of SMILES, complex molecules with many rings and heteroatoms are more difficult to sample.

**Electronic supplementary material:**

The online version of this article (10.1186/s13321-019-0341-z) contains supplementary material, which is available to authorized users.

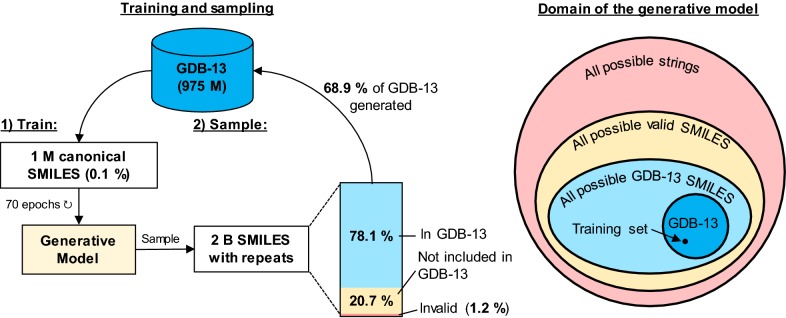

## Introduction

Finding novel molecules with specific properties is one of the main problems that drug discovery faces. One of the most common approaches to this is to explore chemical space by enumerating large virtual libraries, hoping to find a novel region of space containing useful structures. However, the drug-like chemical space is intractably large and a rough estimate would be at least 10^23^ molecules [1]. There are two classical approaches to exploring chemical space. One is to use implicit models, which do not store all molecules in a region of the chemical space but instead represent molecules indirectly. Techniques such as chemical space navigation by mutations [2] or creating reaction graphs have proven to be successful [3, 4]. The other more common way is to use explicit models. By searching public databases that contain molecules obtained from various sources, e.g. ChEMBL [5], new molecules of interest can be discovered. An alternative approach is the GDB project, a set of databases that exhaustively enumerate a part of the chemical space. For example, GDB-13 [6] and GDB-17 [7] are large databases that hold large amounts of drug-like molecules up to 13 and 17 heavy atoms (~ 10^9^ and ~ 10^11^ molecules) respectively. Additionally, GDB-4c [8] is a database that enumerates all possible ring systems up to four rings. These databases include a wealth of novel structures of potential interest for drug discovery [9].

In recent years deep learning has been a major addition in machine learning. Problems that were difficult to tackle before are now successfully approached using deep learning, such as image classification [10], face recognition [11] or playing Go [12]. Recently there has been another step forward in the field with deep generative models, which generate content similar to that upon which they have been trained. Deep generative models have been successfully applied to music composition [13], image generation [14] and language translation [15]. These new methods are also being applied to chemical space exploration in a novel way [16]. When trained with a small subset of molecules, these models generate molecules similar to the training set. Different types of neural networks such as variational auto-encoders (VAE) [17], recurrent neural networks (RNNs) [18, 19] and generative adversarial networks (GAN) [20] trained with string representations (SMILES) [21] from ChEMBL have proven to be successful at generating novel chemical space.

Despite the results obtained by previous research, the question as to how much of the chemical space surrounding the molecules in the training set can be generated by a RNN trained with SMILES remains unanswered. The Fréchet ChemNet distance, [22] which compares a given generated chemical library with real molecule data from ChEMBL, [5] PubChem, [23] and ZINC [24] was recently proposed as a benchmark. However, we think that this metric is not able to unequivocally measure the learning capabilities of a generative model architecture, as it gives information on how likely a generated molecule set is to a set of real bioactive molecules.

**Fig. 1:**
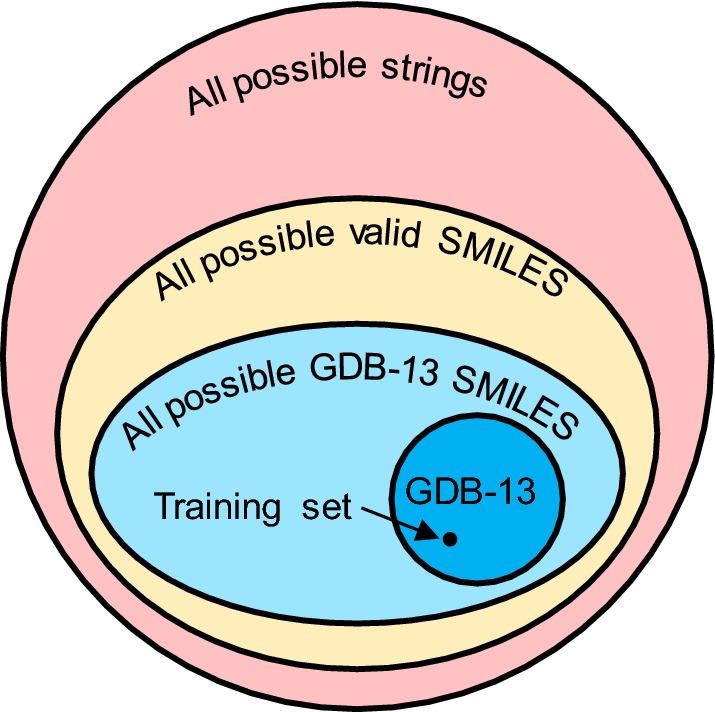
Representation as an Euler diagram of the domain of a RNN trained with SMILES strings. The sets are the following, ordered by their size: All possible strings generated by an RNN (red), all possible valid SMILES (yellow), all possible SMILES of GDB-13 molecules (light blue), all canonical SMILES of GDB-13 molecules (dark blue) and the training set (black). Note that the relative sizes of the different subsets do not reflect their true size

Here we aim to gain insight on how a RNN explores the chemical space and how the SMILES format affect it by training RNNs with canonical SMILES sampled from the GDB databases. We use GDB-13, because this database has denser representation of a reduced chemical space (drug-like molecules up to 13 heavy atoms) and because it has a large yet still manageable size (975 million molecules). Figure 1 illustrates the whole domain of possible outcomes from a RNN trained with SMILES. This domain changes during the training process: before training the RNN generates random strings, a few of which are going to be valid SMILES. After training, the generated strings are mostly valid SMILES that, to a large extent, belong to GDB-13. By computing how much of the whole GDB-13 a model can generate from a small subset and which molecules outside of the domain of GDB-13 are generated, the learning limitations are assessed. To do this, the results obtained from the trained model are compared to those from an abstract ideal model which generates all GDB-13 molecules with uniform distribution. Any model, regardless of its architecture or input format, trained with a subset of GDB-13 can be compared to this ideal model in the same manner, thus creating a new way to benchmark the limitations of models prior to using them to explore chemical space.

Deep learning based molecular generation methods can be applied either to optimize an already existing chemical series or to find through scaffold hopping a completely novel chemical series. While for optimizing a chemical series, it is only necessary to investigate the local chemical space around the series, for scaffold hopping it is important to span the whole desirable chemical space and in addition, not waste time generating molecules outside the desirable domain. Therefore, the proposed benchmark will be especially important for scaffold hopping to ensure that the model explores as much of the desired chemical space as possible, while minimizing sampling undesirable compounds.

## Methods

### Recurrent neural networks

A (feed-forward) neural network [25] (NN) is a machine learning architecture that maps a given input to some output result. After training with a set of predefined input–output examples (called the training set), the system modulates the outputs depending on the inputs given, having a similar behavior to the training set. The internal structure of the system is formed by a series of fully interconnected layers (formed by nodes), starting with the input layer, the hidden layers and ending with the output layer. This topology vaguely resembles a biological neural network, thus its name.

Recurrent neural networks [25] (RNNs) add additional complexity to the feed-forward ones, by converting the topology to a directed graph (which can have cycles). This allows the network to perform recursion and exhibit dynamic temporal behavior. This dynamic behavior creates persistence in the network, not dissimilar to memory. Importantly, a difference between RNNs and NNs is that, instead of having fixed-length input and output vectors, they can be run sequentially. This allows networks to operate on sequences of inputs and thus enables efficient parsing of content of varying length (one-to-many, many-to-one or many-to-many inputs-outputs).

The most common architecture used in RNNs is to connect layers with time-dynamic behavior to layers that normalize the input and the output to achieve an iterative behavior. For each iteration, the model receives two inputs: a vector of numbers and also a hidden state matrix (which contains information from the previous steps) and returns two outputs: an output vector and an updated hidden state matrix. For the next iteration the output and the hidden state from the previous iteration is input. This is repeated until all the input sequences are added, or when the end conditions are met (i.e. outputting specific data).

Since the development of RNNs [26], the system was often unable to learn correctly when many recurrent layers were connected together or the input sequence was too long, due to problems such as vanishing and exploding gradients [27]. These were mitigated by using a very specific layer called a long short-term memory [28] (LSTM). Further research led to the gated recurrent unit [29] (GRU), which has been demonstrated to produce similar results at a lower computational cost.

### Training a model with SMILES

SMILES were discretized into tokens before inputting them to the RNN. Each atom was extracted as a token, taking special care with the multi-letter atoms “Br” or “Cl”. Moreover, all atoms between brackets, such as “[N+]” and “[O−]” were converted into only one token. The set with all the possible tokens is called the vocabulary.

After gathering the vocabulary, two special symbols were added: “^” and “$”, which represent the beginning and end of a sequence respectively. SMILES strings were then encoded using a series of one-hot vectors, each with as many binary positions as tokens in the vocabulary. The represented token having a “1” and the rest “0”. All the SMILES strings were encoded as a matrix with a “^” and “$” token added in the first and last position respectively.

**Fig. 2:**
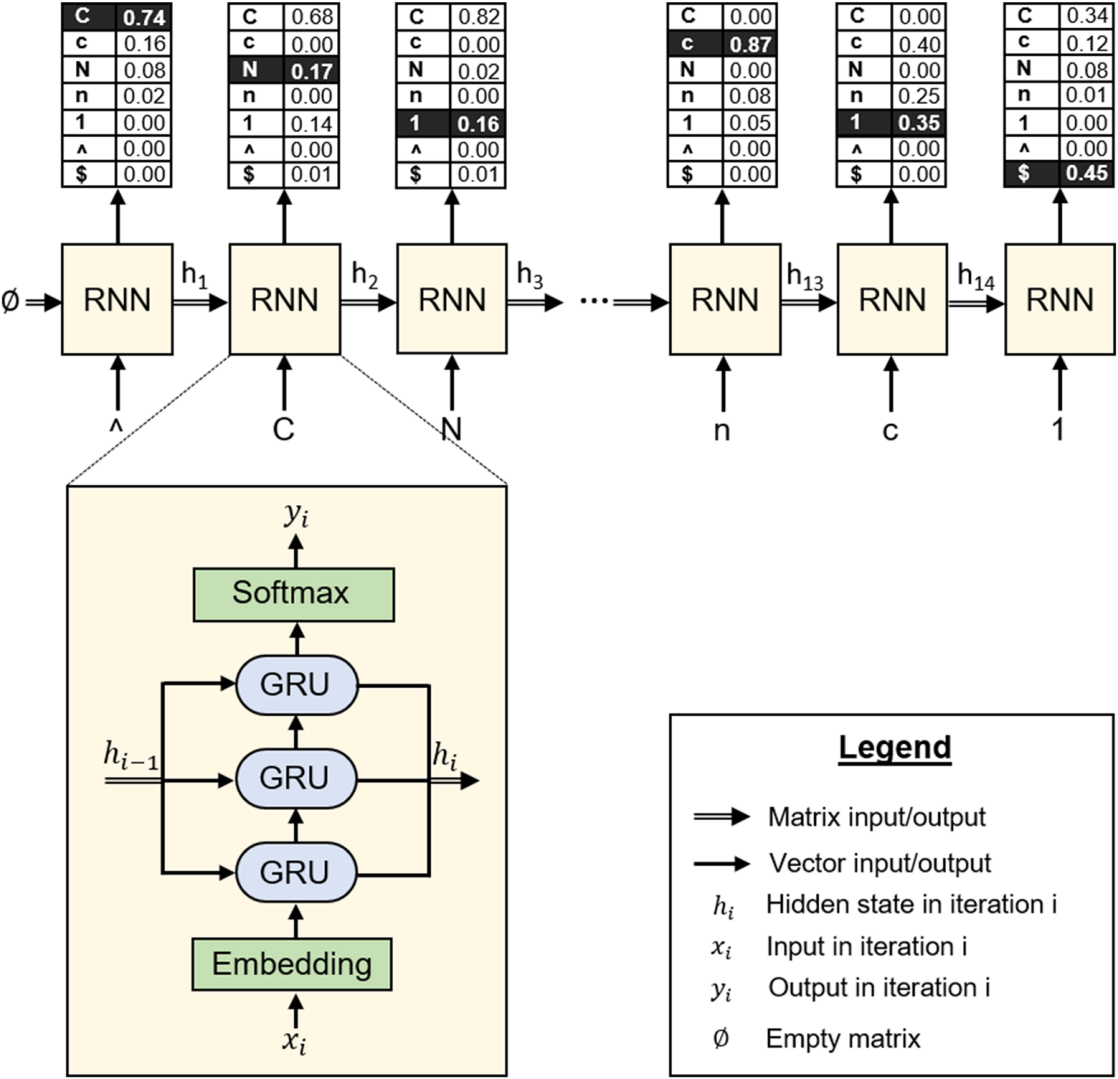
Example of a forward pass of nicotine (CN1CCCC1c1cccnc1) on a trained model. The symbol sampled from the probability distribution at the step $i$ (highlighted in black) is input at the step $i + 1$. This, with the hidden state (h_i_), enables the model to have time-dynamic behavior. Note that sometimes tokens with lower probability are sampled (like in step 1) due to the multinomial sampling of the model. Also note that the probability distributions are not from real trained models and that the vocabulary used throughout this publication is much bigger

The RNN architecture (Fig. 2) used in this publication is similar to previous approaches [18, 19]. First an embedding layer [30] with 256 dimensions converts the discrete one-hot-encoded SMILES to a continuous representation. Then three layers composed of 512 GRU units comprise the bulk of the network. Lastly, a fully-connected linear layer reshapes the output to the size of the vocabulary and a *softmax* operation is performed, making the values sum up to one so they can be used as a probability distribution with the same size as the vocabulary.

For each RNN, two sets were collected beforehand. The training set is a 1 million molecule random sample of GDB-13 used to train the model. Its size was chosen based on what was used in previous research about RNN SMILES generative models [18, 19]. The validation set is another sample of 100,000 molecules not from the training set, used to evaluate the performance of the model during training.

The sampling process of the model is illustrated in Fig. 2. First the “^” token is passed in and the RNN outputs a probability distribution for all the possible tokens. For the next token to be sampled, the RNN requires the previous token and hidden state (memory) to be inputted again. The process continues until a “$” symbol is outputted. Defining $$P\left( {X_{i} = T_{i} |X_{i - 1} = T_{i - 1} , \ldots ,X_{1} = T_{1} } \right)$$ as the probability of sampling token $$T_{i}$$ on step $$X_{i}$$ after having sampled tokens $$T_{i - 1} \ldots T_{1}$$ on steps $$X_{i - 1} \ldots X_{1}$$, the resulting probability on step $$i$$ is:

$$P\left( {X_{i} = T_{i} , \ldots ,X_{1} = T_{1} } \right) = P\left( {X_{1} = T_{1} } \right) \cdot \mathop \prod \limits_{k = 2}^{i} P\left( {X_{k} = T_{k} |X_{k - 1} = T_{k - 1} , \ldots ,X_{1} = T_{1} } \right)$$

As the value would rapidly diminish to 0, due to hardware precision problems, (natural) logarithm sums are used:

1 $$NLL_{i} = - ln P\left( {X_{i} = T_{i} , \ldots ,X_{1} = T_{1} } \right) = - ln P\left( {X_{1} = T_{1} } \right) - \mathop \sum \limits_{k = 2}^{i} ln P\left( {X_{k} = T_{k} |X_{k - 1} = T_{k - 1} , \ldots ,X_{1} = T_{1} } \right)$$

This value is called a negative log-likelihood (NLL) and it gives a measure on how likely a sequence is to appear when randomly sampling the model. Its range is $\left[ {0, + \infty } \right)$ with higher values corresponding to lower probabilities.

As in previous research [18, 19], backpropagation with the ADAM optimizer was used to train the RNN. The goal is to minimize a cost function $J\left( w \right)$ for all molecules in the training set. To achieve that, it calculates from the last to the first step the average of the $J\left( w \right)$ of a set of sequences (a batch). From this, a gradient is calculated which can be used to iteratively fit the model to the training data. Formally, the loss function is the partial NLL up to position $i$:

$$J\left( w \right) = NLL_{i}$$

The teacher’s forcing [31] method was used. In this method the likelihood calculation on step $i$ is calculated from the previous tokens in the training SMILES and not from the possibly wrong token of the untrained RNN. This allows the RNN to learn the information in the training set faster and more reliably.

The training data was passed to the RNN multiple times: each iteration, called an epoch, all compounds in the set were input to the RNN. To enhance the learning process learning rate (LR) decay was used. This hyperparameter controls the optimization speed of the learning process, higher LRs imply faster learning but less refined solutions. After some early testing it was observed that a LR greater than 10^−3^ and smaller than 10^−5^ have no effect on the training whatsoever, so the LR changes from 10^−3^ to 10^−5^, being multiplied by a constant every epoch.

### Ideal model

In our research, a RNN-based model must learn how to generate SMILES and how to create molecules that appear in GDB-13. An ideal model is an abstract model that samples molecules from GDB-13 and only from GDB-13. Formally, the probability of sampling any molecule in the ideal model follows a uniform probability distribution with $p = \frac{1}{{\left| {GDB - 13} \right|}} = 1.02 \cdot 10^{ - 9}$. Due to the probabilistic nature of RNNs, no trained model will be able to have the same behavior, thus an ideal model serves as an upper bound.

We can calculate the expected number of times GDB-13 needs to be sampled to obtain 100% of the database. This problem is commonly known in mathematics as the “coupon collector problem” [32]. It was originally used to calculate the number of coupons (or stickers) that are needed to be bought to be able to obtain the full collection, knowing that every time a coupon is bought it is sampled with replacement from a distribution containing all possible coupons. Formally, for a uniform distribution with $n > 1$ coupons:

2 $$E\left[ {T_{u} } \right] = n \cdot H_{n} \approx n\left( {\ln \left( n \right) + \gamma } \right) + \frac{1}{2}$$

where $H_{n}$ is the n-th harmonic number and $\gamma$ is the Euler–Mascheroni constant. By fitting this to the GDB-13 we would need to sample on average 20,761,554,747 SMILES. For non-uniform probability distributions, this expected value is a lower bound and it tends to infinity for distributions where $\exists p_{k} \to 0$ (Additional file 1: Suppl. Material S1). Sampling the GDB-13 20 billion times is a computationally expensive task, so we can also obtain the expected fraction of a collection with $n > 2$ coupons if $k > 1$ were sampled from the ideal model (Additional file 1: Suppl. Material S2):

3 $$fraction\_uniform = 1 - \left( {1 - p} \right)^{k}$$

In the case of a sample of $k = 2 \cdot 10^{9}$ molecules from the ideal model the average fraction of molecules sampled would be $1 - \left( {1 - 1.02 \cdot 10^{ - 9} } \right)^{{2 \cdot 10^{9} }} = 0.8712$. This value is an upper bound (Additional file 1: Suppl. Material S3): any model that is either non-uniform or non-complete will have a smaller fraction of molecules from GDB-13. This allows us to measure the completeness and uniformness of any generative model architecture trained with GDB-13.

### Sampling SMILES from a model

To be able to evaluate how much of GDB-13 can be reliably sampled from a model, it must be sampled at least 20 billion times (Eq. 2). This has an unfeasible computational cost. For this reason, samples of 2 billion molecules were performed, which account for approximately 10% of the optimal sample size. After each sample, several tests were done: the database was checked for duplicates, for invalid SMILES, for non-canonical SMILES and was intersected with GDB-13, yielding 2 subsets: IN and OUT of GDB-13.

### PCA plots with MQN

PCA plots were based on the method described previously in literature [33]. The 42-dimension MQN fingerprint [34] was calculated with the JChem Library 18.22.0 from ChemAxon (www.chemaxon.com) for each of the molecules in the dataset. Then, without any normalization or standardization, a principal component analysis (PCA) was performed on the 42-dimensional resulting dataset. The two first principal components were selected and normalized to values between 0 and $w$ or $h$ and molecules were organized in buckets. Each bucket represents a pixel $\left( {x,y} \right)$ in the resulting $w \times h$ plot with a black background. A descriptor was also calculated for all molecules in each bucket and the average and count were calculated and normalized to the range $\left[ {0,1} \right]$. To color the pixels, the hue-saturation-value (HSV) format was used with the normalized average descriptor as hue, a fixed value of 1.0 as the saturation and $value = \hbox{min} \left( {0.25,\log_{10} (count_{norm} } \right)).$ With this setup, the pixels that have low count are gradually merged with the background and those that have the highest counts stand out.

### Labelling sampled molecules out of GDB-13

Sampled molecules not included in GDB-13 were labeled with the topological and chemical filters used in the enumeration process of GDB-13 that they broke [6, 35]. The molecules with disallowed topology were labelled the following way: carbon skeletons from all the molecules in GDB-13 were calculated and compared to the carbon skeletons for each sampled molecule, labelling the molecules whose skeleton was not in GDB-13. All tautomers for all the molecules were calculated with MolVS [36] 0.1.1. For each molecule, if one tautomer was part of GDB-13, the molecule was labelled as a tautomer. Molecules with disallowed functional groups, heteroatom configurations or bonds were detected using SMARTS.

### Technical details

All the programming, except noted, was done in Python 3.6 using RDKit [37] version 2018.03 as the chemistry toolkit and PyTorch [38] 0.4.1 as the deep learning library. Stochastic gradient descent was used for training with the ADAM [39] optimizer with parameters $\beta_{1} = 0.9, \beta_{2} = 0.999, \varepsilon = 10^{ - 8}$ and a batch size of 128.

The GDB-13 database was obtained from the gdb.unibe.ch website and preprocessed with RDKit to obtain canonicalized SMILES and to filter molecules impossible to read with the toolkit. The final size of the database was 975,820,187 molecules. Data processing and PCA calculation were done with Apache Spark [40] 2.3.1 and all datasets were stored in Apache Parquet files. All plots, including the PCA maps, were created with Matplotlib [41] and Seaborn [42]. The Jensen-Shannon Divergence was calculated with an in-house script using SciPy [43]. Table 1 shows the resources and cost of the different computations described previously, all of which were performed in CentOS 7.4 with Tesla V-100 (Volta) graphics cards and CUDA 9.

**Table 1:** Computational resources and cost associated with training and sampling the model and annotating a 2B sample

Operation	CPUs	RAM	GPU	Time
Training a model	4	32 GB	1	8 min/epoch
Sampling molecules	4	32 GB	1	33 million/h
Annotating 2B molecules	32	256 GB	0	24 h

## Results and discussion

### Using negative log-likelihood plots to guide the training process

**Fig. 3:**
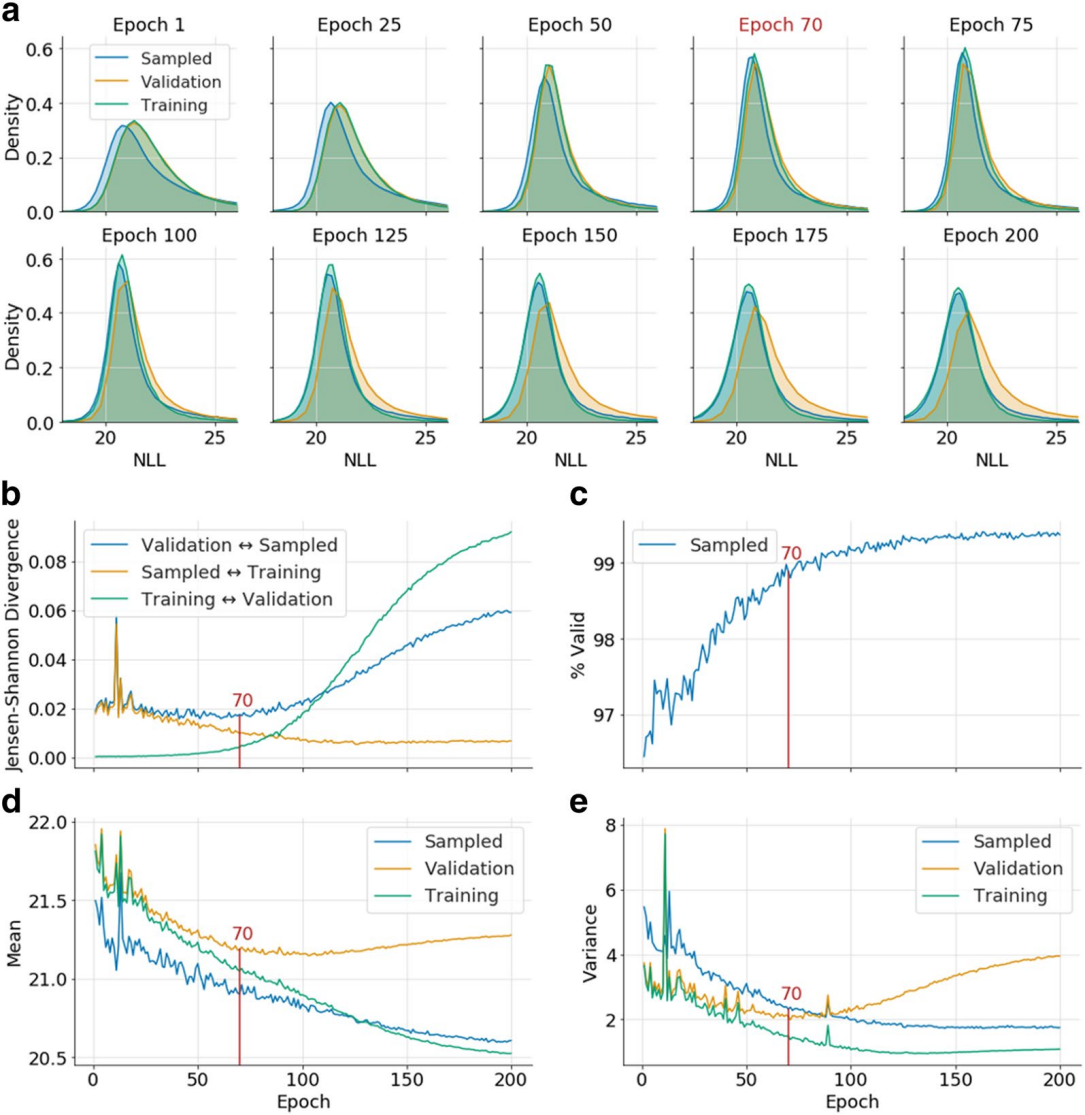
Metrics used to evaluate the training process. The red line at epoch 70 represents the chosen epoch used in further tests. The negative log-likelihood (NLL) is calculated with natural logarithms. **a** 10 NLL plots of the training, validation and sampled sets every 25 epochs (from 1 to 200) and the chosen epoch (70). **b** JSD plot between the three NLL distributions from the previous section for each of the 200 epochs. **c** Percentage of valid molecules in each epoch. Notice that the plot already starts at around 96.5%. Mean (**d**) and variance (**e**) of the three distributions from section (**a**). Note that spikes around epochs 1–20 are statistical fluctuations common in the beginning of the training process of a RNN, when the learning rate is high

A model was trained with a set of 1 million compounds randomly obtained from GDB-13. An initial way to assess the quality of the sampled molecules from the trained model is to check the percentage of valid molecules (Fig. 3c). This metric has often been used to train models [18, 19], but in the case of the GDB databases it proves to be insufficient, as it is always over 96.5%. To view the progress of the training, negative log-likelihoods (NLLs) of the SMILES in the training, validation and sampled sets were calculated after training the model each epoch. These NLLs were plotted together as histograms every 25 epochs (Fig. 3a). Also, the Jensen–Shannon divergence (JSD) of all pairs of NLL plots was calculated (Fig. 3b). This measure allows the quantification of the differences between each pair of distributions.

Figure 3 plots are interpreted as follows: after epoch 1, the sampled set NLL distribution has the lowest average (higher probability) and the other two sets are extremely similar and have a higher NLL (lower probability). This means that the model is not completely trained, as the SMILES strings sampled are only a subset of the ones in the training set. Between epochs 25–50, the distributions become more similar, and around epochs 50-100 the three plots match as much as possible, as can be seen both in (a) and in (b). When all the plots are similar it is equally probable to sample a SMILES from the training set as it is a SMILES outside it, implying that a higher percent of the database can be sampled. After this, the training set NLL distribution becomes more similar to the sampled set while the validation set has higher NLL. This indicates that the model is gradually being over trained, as a molecule from the training set will be sampled from it with a higher probability than a molecule from the validation set. This trend becomes more pronounced in later epochs.

To further discern whether the model is uniform and complete, the mean ($\mu$) and especially the variance ($\sigma^{2}$) of the NLL distributions have been calculated after each training epoch (Fig. 3c, d). Knowing that the uniform model NLL plot has $\sigma^{2} = 0$ and $\mu = - \ln \left( {\frac{1}{{\left| {GDB - 13} \right|}}} \right) = 20.7$, the variance and the mean of the validation set should be as similar to these values as possible. Both descriptors reach plateaus at around epochs 60–150 for the mean and 60–90 for the variance.

By comparing all the intervals from the three different plots, we can obtain a joined interval from around epoch 60 to 90, in which the model will have learned how to create the biggest and more uniform domain.

### Sampling the model and analyzing its domain

**Fig. 4:**
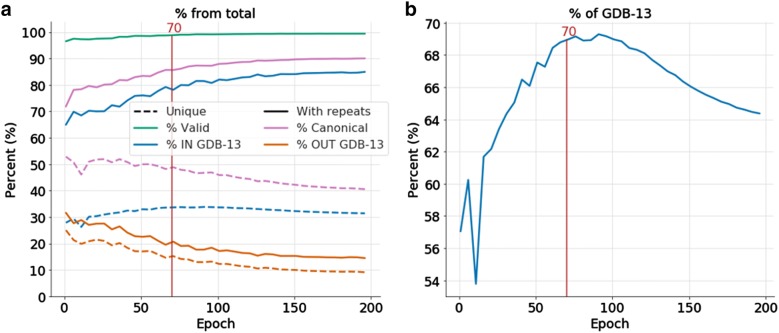
Results from sampling 2 billion SMILES from the 1 M model every five epochs (from 1 to 195). The red line at epoch 70 represents the chosen epoch for further tests. **a** Percent of the total sample (2B) that are valid SMILES, canonical SMILES, in GDB-13 and out of GDB-13. Solid lines represent all SMILES sampled, including repeats, whereas dotted lines represent only the unique molecules obtained from the whole count. **b** Close-up percentage of GDB-13 obtained every five epochs. Notice that the plot starts at around 54% and that the drop around epoch 10 correlates with the training fluctuations already mentioned in Fig. 3

To validate the previous method, 2 billion SMILES strings were sampled every five epochs (totaling 80 billion). As can be seen in Fig. 4, the total percent of generated molecules including repeats that are part of GDB-13 always increases, but in Fig. 4 the percent of unique molecules generated that are in GDB-13 is maximal at epoch 90 (69,2%), but there is a plateau starting around epoch 70 (68.9%) and decreases steadily again after epoch 100 (68.9%). Also, the sampled molecules not included GDB-13 steadily decrease during the whole training. These results are very similar to the results obtained from the analysis of the NLL plots, the mean and the variance plot in Fig. 3b, d, e. Having a model representing a more uniform sampling (epoch 70) conflicts with having a more focused sampling (epoch 100). Depending on the specific needs for a given project a different epoch should be chosen, yet the differences are very small. Epoch 70 was chosen for future experiments with this model, because a more uniform model was desired.

For any molecule there are many SMILES that uniquely represent it. In Fig. 1 the light and dark blue sets represent the number of possible SMILES for all the molecules and only one canonical SMILES for each molecule respectively. In the ideal model, only the canonical SMILES for each molecule are generated. Figure 4 shows (pink) that 85.6% of the SMILES in epoch 70 were generated directly as canonical, implying that the model can learn the canonical form of most of the generated SMILES. Notice also that the number of unique canonical SMILES decreases steadily. This is correlated with the model not being uniform, and this trend is further pronounced after epoch 100, as the molecules from the training set are generated more often.

### Understanding the diversity of the generated molecules

25 models with the same parameters as in the previous section were trained with a different 1 M random sample obtained from GDB-13. The probability of sampling each molecule from GDB-13 is averaged and molecules not generated by any model have a higher chance to be problematic due to the limitations of the model and not by chance.

**Fig. 5:**
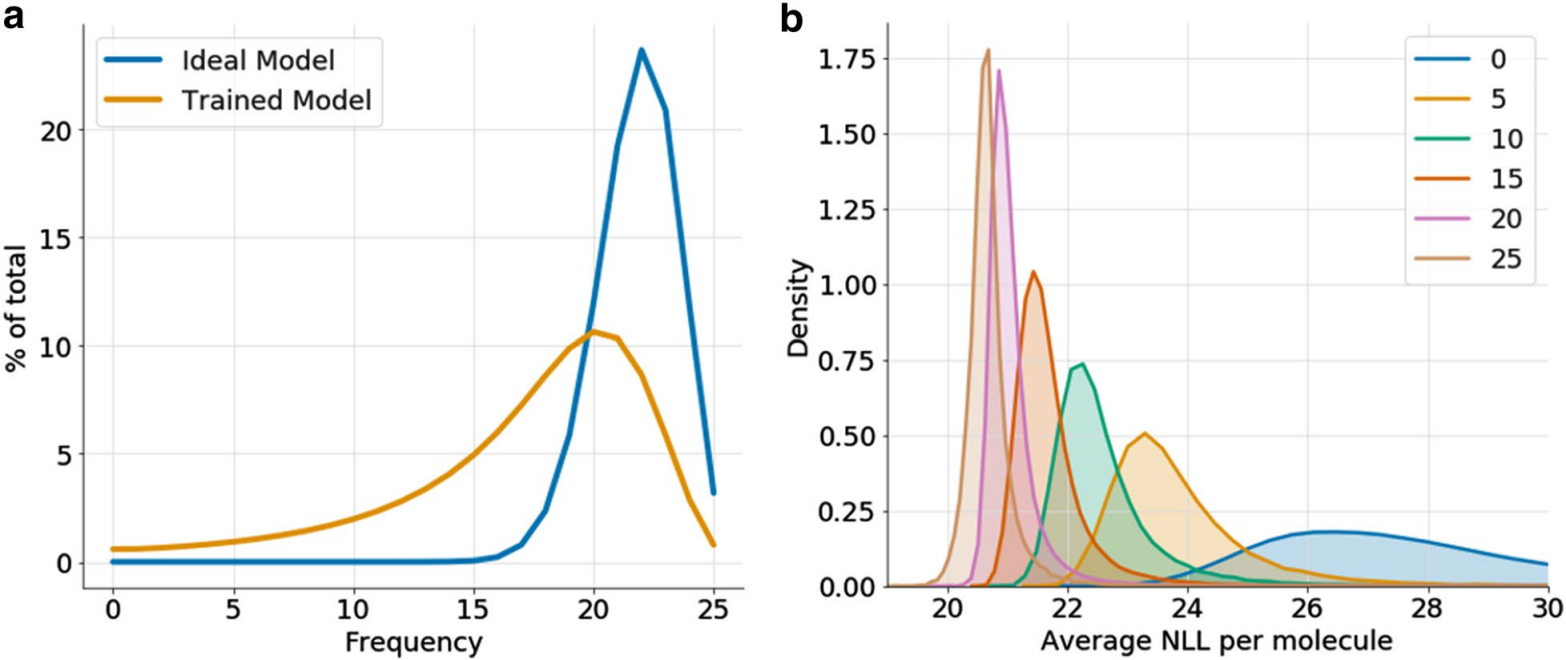
**a** Histograms of the frequency of the RNN models (orange) and the theoretical (binomial) frequency distribution of the ideal model (blue). **b** Histograms of the average NLL per molecule (from the 25 models) for molecules with frequency 0, 5, 10, 15, 20 and 25 computed from a sample of 5 million molecules from GDB-13

For each model a sampling of 2 billion molecules was performed in epoch 70 (summing up to 50 billion molecules), repeated molecules were filtered and the whole sample was separated between molecules contained and not contained in GDB-13. Note that the number of molecules needed to sample from the ideal model to obtain 100% of the database on average is around 21 billion (Eq. 2), much less than the 50 billion molecules sampled in this experiment. The frequency for each molecule in GDB-13 was computed, which is the number of times from 0 (not sampled in any model) to 25 (sampled in all models) each molecule was uniquely sampled from each model. In the ideal model, each sample can be considered as a Bernoulli trial with $p = 0.8712$ ((Eq. 3)), so the distribution of the frequency would follow a binomial distribution with $n = 25, k = 2 \cdot 10^{9}$ and $p = 0.8712$. Figure 5a shows that the two distributions have a different mean (17.1 and 21.8) and mode (20 and 22) and the distribution obtained from the RNN models has an extremely long tail. Moreover, 5,720,928 molecules (0.6%) were never sampled by any model. Notice also in Fig. 5b that frequency is heavily correlated with the average negative log-likelihood for each molecule obtained from every model.

### Analysis of the sampled molecules included in GDB-13

**Fig. 6:**
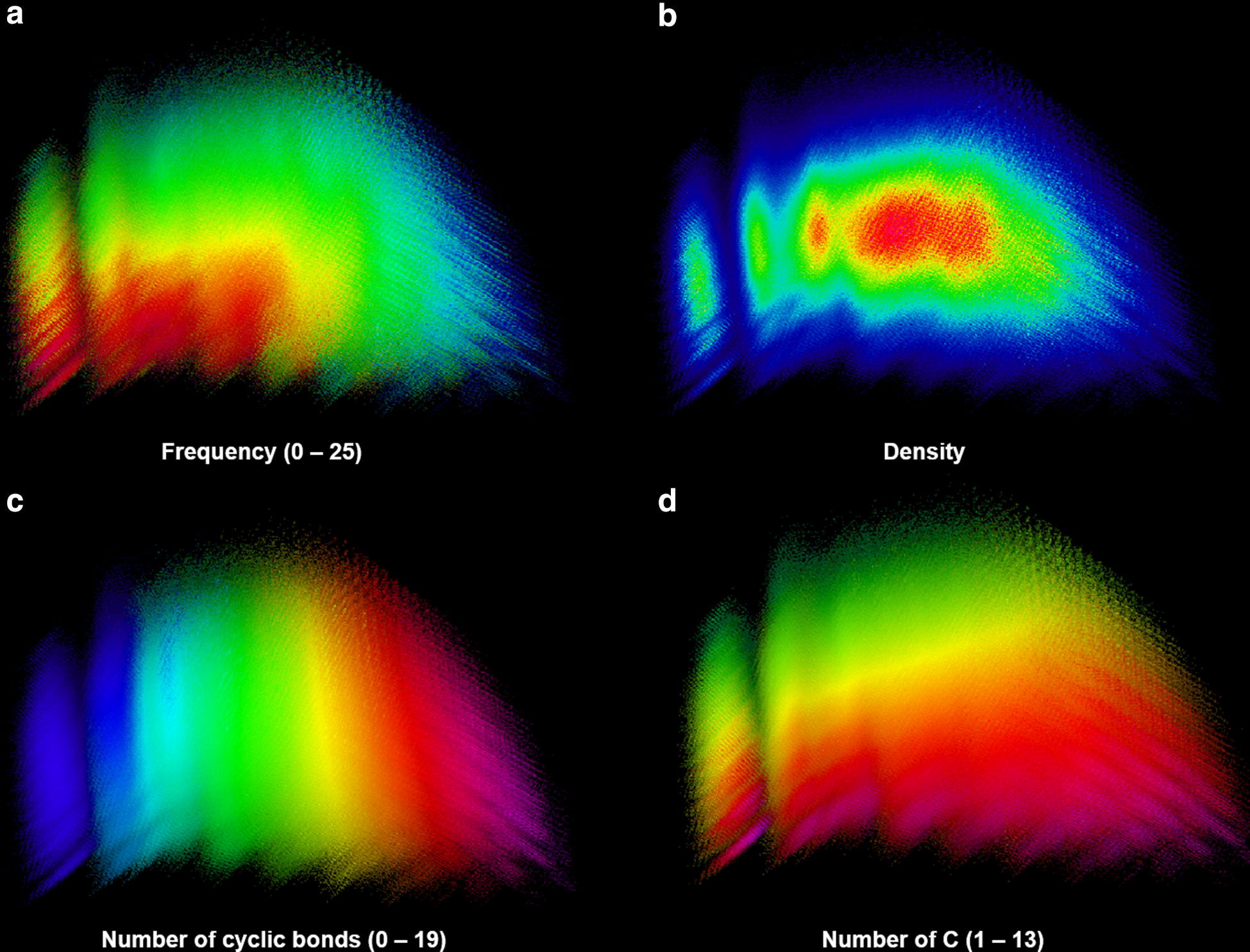
**a**–**f** MQN PCA plots (Explained variance: $PCA_{1} = 51.3 \% , PCA_{2} = 12,2 \%$) calculated from a 130 million stratified sample of GDB-13 with 5 million molecules from each frequency value (0–25) colored by different descriptors. In all plots each pixel represents a group of similar molecules and its color represents the average value of a given descriptor. The colors rank from minimum to maximum: dark blue, cyan, green, yellow, orange, red and magenta. Each plot has the numeric range (min–max) between brackets after its title. Plots are colored by: **a** Number of trained models that generate each molecule. **b** Occupancy of every pixel. **c** Number of cyclic bonds. **d** Number of carbon atoms

PCA plots of the MQN fingerprint were performed with a sample of GDB-13 stratified by frequency (Fig. 6). Figure 6a, shows that there is a difference between the molecules that have lower (top-right) and higher (bottom-left) frequency. Nevertheless, the density plot (Fig. 6b) shows that the most densely packed regions are at the center and occupied by molecules with both a high and a low frequency. Additional PCA plots were generated with some key descriptors that help pinpointing the different regions of the chemical space. Figure 6c shows that pixels at the right have mostly cyclic bonds, implying more rings and fewer sidechains and linkers. This area is mostly covered by molecules that have low frequency. Moreover, Fig. 6d shows that pixels at the top have more heteroatoms. This closely matches the top lighter area in Fig. 6a, which features molecules with low frequency.

From the previous plots, molecules with many heteroatoms or complex topologies have a lower probability of being sampled than molecules with less rings and more carbon atoms. However, Fig. 6b also shows that most of these structures are in lower density zones of the database, which implies that are only a small part of the database. In Table 2, 24 fragment-like molecules with frequency 0, 5, 10, 15, 20 and 25 were selected from GDB-13 and shows that molecules with lower frequency have a tendency to have a more complex structure, especially more cyclic bonds, although it is not possible to separate them clearly.

**Table 2:**
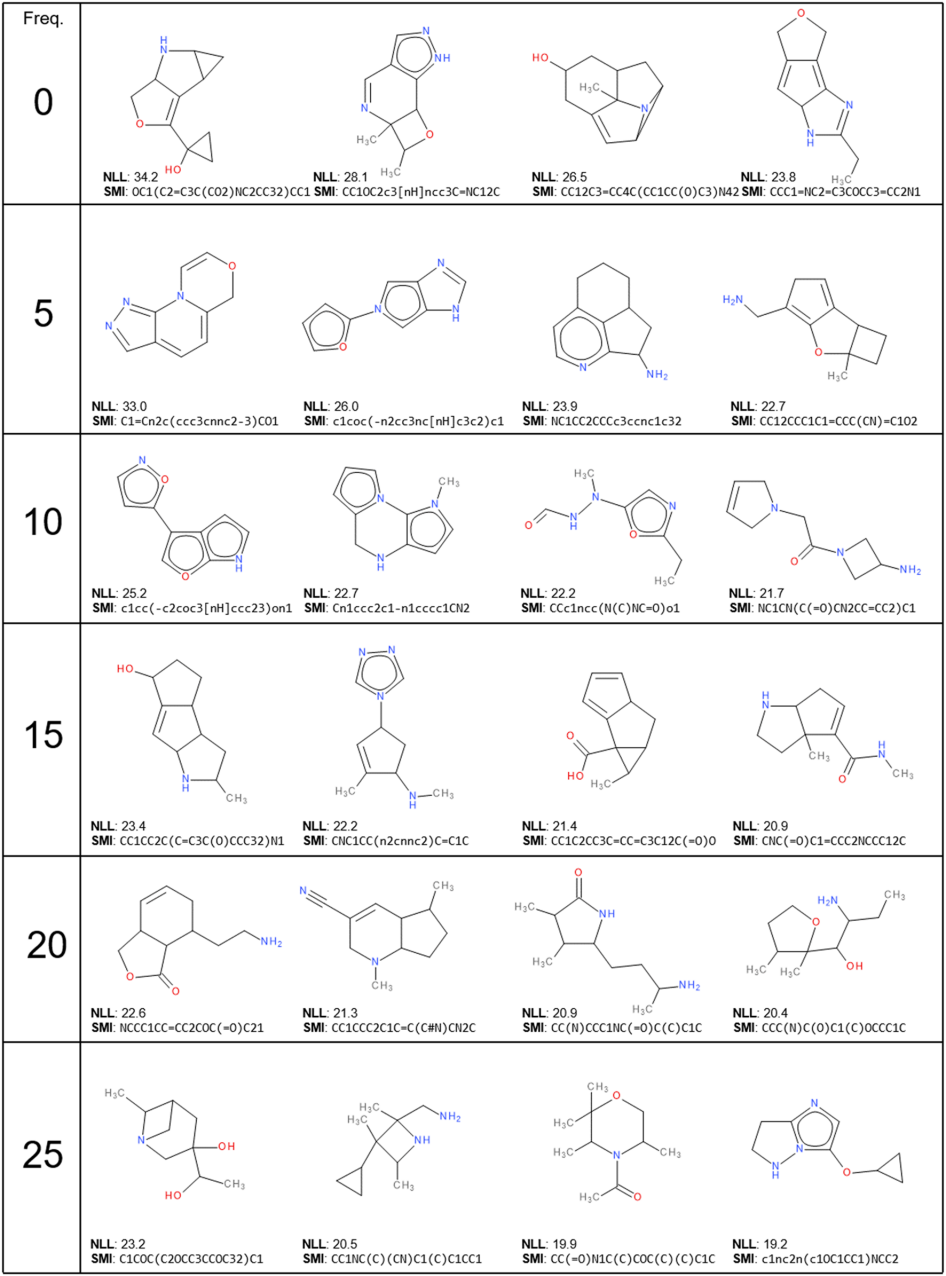
A selection of 24 fragment-like molecules obtained from GDB-13 with frequency 0, 5, 10, 15, 20 and 25. The molecules are sorted top to bottom by frequency and left to right by average negative log-likelihood (NLL) of the 25 models. A random sample of 10 million molecules annotated with the frequency and the average NLL is available for download (http://gdb.unibe.ch/downloads)

**Fig. 7:**
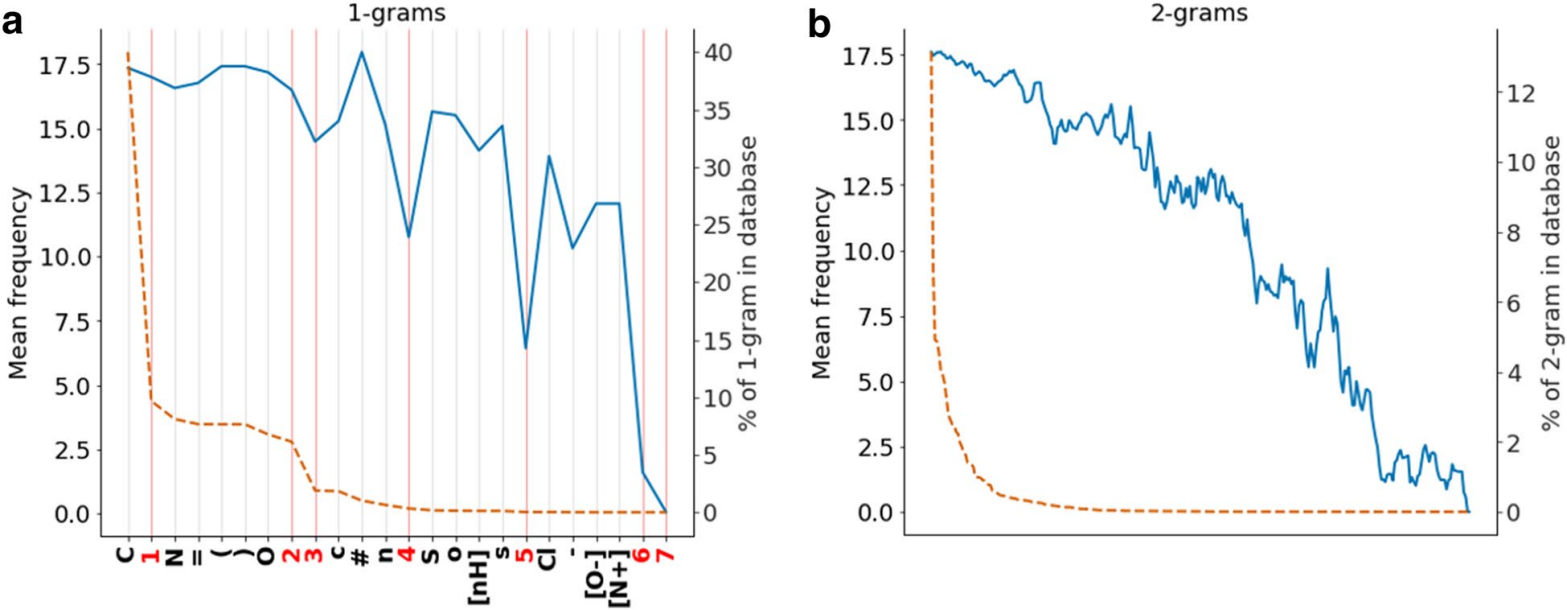
Plots of the frequency (left y axis) and the percent in database (right y axis) of 1 and 2-g in the canonical smiles of all GDB-13 molecules. The plot is sorted by the percentage present in the database. **a** Plot with the 1-g (tokens). In blue the mean frequency and in orange the percent of 1-g in database. Notice that the numeric tokens have been highlighted in red. **b** Plot with the 2-g mean frequency (blue) and percent (dashed orange). As the number of 2-g is too large (287), the x axis has been intentionally left blank and the mean frequency has been smoothed by an average window function size 8

To further understand how molecules are generated, the composition of the SMILES was analyzed. As shown in Fig. 7a (dashed orange line), the 1-g (token) count distribution is exponential and mostly features C (40%). In order, less featured tokens are 1, N, =, (,), O and 2. The rest of the tokens sum up to less than 7% of the total. SMILES representing simple topologies use mostly the tokens enumerated before and molecules that have complex shapes tend to have more rings, so they have less common tokens, such as 3,4, …, 7. The frequency of the molecules containing each token is also plotted in Fig. 7a, showing that the frequency correlates with the counts. Note especially, marked in red in Fig. 7a, the numeric tokens starting from 4 tend to have a significantly lower average frequency than the neighboring tokens. This means that molecules in GDB-13 with four or more rings are significantly less likely to be sampled than others. One explanation is that these tokens only appear in pairs in valid SMILES, which indicates that learning how to create a correct molecular SMILES with these tokens is much more difficult than with other equally frequent tokens, as both tokens in each pair must be correctly positioned with respect to each other. Additionally, molecules in GDB-13 (max. 13 heavy atoms) with more than three rings have extremely complex topologies. When performing the same analysis for 2-g same interpretation applies (Fig. 7b): the count is correlated with the average frequency and the most frequent 2-g (CC, C1, C(,)C, C=) match the SMILES of simple molecules and the less frequent (5o, 3[N+], 7O, 7(, 72) match exclusively molecules with complex topologies and several rings. This implies that the n-grams that appear fewer times in the database, i.e. in the training set, are not learned correctly and thus have a lower probability of being sampled. Therefore, molecules that contain an increasing number of low probability n-grams in their canonical SMILES, will progressively have a lower probability of being sampled (Eq. 1).

### Analysis of the sampled chemical space outside of GDB-13

**Fig. 8:**
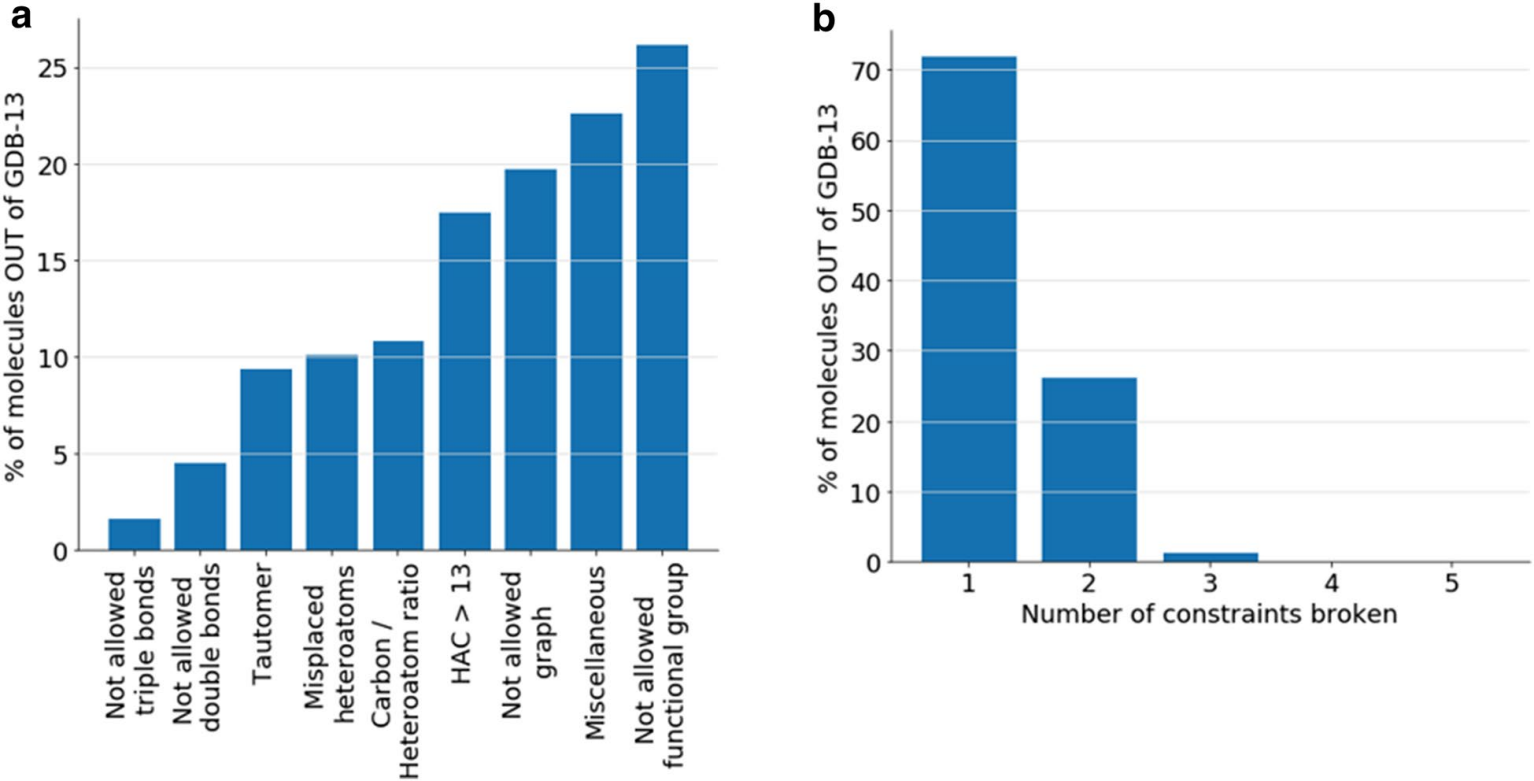
Distribution of a sample of 3 million molecules obtained from all the outside of GDB-13 sampled by the RNN model. **a** Histogram of the GDB-13 constraints broken by each molecule. Notice that a molecule can break more than one constraint. **b** Distribution of the number of GDB-13 constraints broken by each molecule

All the SMILES outside of GDB-13 generated by the 25 models were joined obtaining a database with 10,084,412,477 molecules. After filtering repeated molecules, a set with 2,979,671,366 unique molecules was obtained, from which a sample of 3 million was used for further research. Each molecule was then labelled with the constraints used to enumerate GDB-13 [6, 35] that it breaks (see methods). Figure 8 includes a plot with the percent of molecules that break each constraint (Fig. 8a) and another histogram with the number of constraints broken per molecule (Fig. 8b). The most common broken constraint, not allowed functional groups (26.2%), is the most complex one to learn, as any given functional group can have multiple SMILES strings, depending on where it is positioned in the molecule, thus making it more difficult to learn the string patterns to avoid. Also, 19.8% of the molecules have a graph that was filtered during the GDB-13 enumeration process, which correlates with the problems encountered when generating molecules with complex graph topologies: the model is not able to correctly learn the underlying graph topologies of the molecules. Additionally, due to the probabilistic nature of the model, 17.5% of the molecules generated outside of GDB-13 have more than 13 heavy atoms. Heteroatom/Carbon ratios used to create GDB-13 are generally followed (10.9%) and there are a similar number (10.1%) of molecules with disallowed neighboring heteroatom configurations. These constrains can easily be learnt by the model, as they have very little topological complexity compared to the previous two. For the same reason, 9.4% of the database are tautomers of molecules existing in GDB-13 and less than 7% of the molecules have problems with double or triple bonds. Interestingly, the miscellaneous category (22.7%) includes all molecules that are not in GDB-13 and that have broken none of the previous constraints. This occurs partially due to compatibility issues with the chemical library used (GDB-13 was created with JChem from 2008 and this research uses RDKit from 2018) and because GDB-13 is not completely exhaustive. The enumerative process used to create GDB-13 performed several levels of filtering: when a molecule was filtered out in an intermediate step, the molecules that would have derived from it were never generated. Most of these molecules would have probably been filtered if they had been generated, as they are extremely uncommon. Lastly, Fig. 8b shows that 72% of the molecules only break one constraint, hence the chemical space generated outside of GDB-13 is very similar to the space represented by GDB-13.

### Counteracting the limitations in models using SMILES

The previous two sections show that the SMILES format adds two substantial biases to the chemical space learned by our model. Firstly, the model has more difficulties generating molecules with many rings, especially with complex ring systems. This limitation stems from the nature of SMILES: highly cyclic molecules have longer SMILES sequences than equally-sized acyclic molecules and the relative positioning of the ring tokens is context-sensitive. Fortunately, most drug-like molecules (like those in ChEMBL) tend to have simpler structures than GDB-13 molecules, making this problem less important for generative models that target the known drug-like space. One way that could help overcoming this bias is to carefully tailor the training set to feature more molecules that include complex ring systems. This will give the model more examples of complex molecules from which to learn, even though it would possibly add other biases. Also, a theoretical approach that could help was recently published [44] and alters the SMILES semantics, making ring tokens behave differently. This approach may make some ring systems have a less convoluted syntax but could make the SMILES syntax significantly more complex for the model to learn. The second bias has to do with the molecules outside of GDB-13 being incorrectly generated by the model and is also partially associated with the SMILES syntax. For instance, there are many ways of writing SMILES strings that represent most functional groups and there are many molecules with extremely different SMILES that share the same underlying graph. These ambiguities make it especially difficult for the model to learn to correctly filter some molecules that have not allowed functional groups or graphs. One way that we think it could partially mitigate these problems is using a less ambiguous molecular representation that also separates the graph from its decoration such as graph generative models [45].

### Training models with smaller training sets

Another important question is how using smaller datasets (100.000 molecules or less) would impact the chemical space generated. We performed some preliminary analysis, and we found that models with smaller training sets tend to overfit more and faster. Due to the reduced amount of diversity present in them, the model easily learns to reproduce the training set. That is why training models with smaller subsets of the GDB-13 database could give us information on which are the best architectures and hyperparameter configurations to minimize the overfit and optimize the learning capabilities of any model.

## Conclusions

This study shows that a large amount of chemical space can be sampled with generative models that are trained only with a very small sample of that chemical space. Specifically, we generate up to 68.9% of GDB-13 by using a training set with only 0.1% of the database. The model is not only capable of learning basic chemistry (e.g. valency, ring structures) but also to follow complex constraints applied during the GDB-13 enumeration process, such as heteroatom ratios and positioning of double and triple bonds. More difficult constraints, e.g. complex graph topologies or not allowed functional groups are more difficult to learn mostly due to the limitations of the SMILES notation. We developed a computationally efficient method to monitor and assess the quality of the training process using NLL plots. This method allows to identify the different stages of the training process and select a better model. To further understand the NLL plot analysis, we sampled the model every five epochs and compared the results with those from the ideal model. Moreover, this sampling can be used as a benchmarking tool for molecular generative model architectures as we showed that the ideal model sets an upper limit (87.12%) to the amount of GDB-13 generated with a 2 billion sample. We encourage researchers to try training models with different architectures or input formats on GDB-13, sample them 2 billion times, calculate the coverage and compare the results. This may lead to a better understanding of the different architectures of molecular generative models. Finally, we performed an extensive analysis to find if there is any bias attributable to the model using the generated chemical space from a joined sample of 25 models at the same epoch. We obtained that although most of the problematic molecules have a tendency of having more cyclic bonds and heteroatoms, the main difference arises from issues within the SMILES syntax, especially related to using numeric ring tokens. We think that all the methods described here will help to find new generative model architectures that can overcome some of the limitations of the current ones.

## Additional file

**Additional file 1.** Supplementary material.

## Abbreviations

ADAM: adaptive moment estimation
GAN: generative adversarial network
GDB: generated database
GRU: gated recurrent unit
HSV: hue–saturation–value
NLL: negative log-likelihood
LR: learning rate
LSTM: long short-term memory
MQN: molecular quantum numbers
NN: neural network
PCA: principal component analysis
RNN: recurrent neural network
SMARTS: smiles arbitrary target specification
SMILES: simplified molecular-input line-entry system
VAE: variational auto-encoder
