# Supplementary material for: Exploring the GDB-13 chemical space using deep generative models
Source: J Cheminform. 2019 Mar 12;11:20. doi: 10.1186/s13321-019-0341-z (PMC6419837; doi:10.1186/s13321-019-0341-z)
Supplement: Supplementary file 1 — Additional file 1. Supplementary material. [file 13321_2019_341_MOESM1_ESM.docx]

Exploring the GDB-13 chemical space using deep generative models

Josep Arús-Pous†⊥*, Thomas Blaschke†║, Silas Ulander§, Jean-Louis Reymond⊥, Hongming Chen†, Ola Engkvist†

Supplementary material

**S1: Generalization of the “coupon collector problem” to non-uniform distributions**

The expected number of times that a uniform distribution needs to be sampled with replacement to obtain the complete domain (Equation 2) can be easily generalized to a non-uniform distribution, having the previous equation as a lower bound of the expected value:

Given $p_{k}\leq p_{k+1} \forall k\in\left[ 1,n \right]$ we define:

$$N=\sum_{k=1}^{n} \frac{p_{i}}{p_{1}}$$

(Suppl. Equation 1)

Notice that $N\geq n$ and that each $p_{i}$ is subdivided into many smaller buckets, all of them of the smallest $p_{i}$. This allows the conversion from a non-uniform to a uniform distribution. Hence, the upper bound for the expected value is:

$$E\left[ T_{nu} \right]<N\cdot H_{N}\approx N\left( \ln\left( N \right)+\gamma\right)+\frac{1}{2}$$

(Suppl. Equation 2)

The expected value can tend to infinity for distributions where ${\exists p}_{k}\to0$. This is the case for all the trained models in this publication.

**S2: Expected fraction of unique coupons on a collection with size *n* when *k* coupons are sampled with replacement**

Given a non-uniform probability distribution where $\sum_{i=1}^{n} p_{i}=1$, Let $X$ be the number of different coupons collected when sampling $k>1$ times a set with $n$ coupons. The probability of sampling $k$ times without picking any specific coupon is ${P(t}_{i})={(1-p_{i})}^{k}$,and $P(\bar{t}_{i})=1-X_{i}$ as the complementary probability. The expected number of different items when sampling $k$ times is:

$$E\left[ X \right]=\sum_{i=1}^{n} P(\bar{t}_{i})=n- \sum_{i=1}^{n} {(1-p_{i})}^{k}$$

(Suppl. Equation 3)

The fraction of the database can then be obtained as:

$$fraction=\frac{n- \sum_{i=1}^{n} {(1-p_{i})}^{k}}{n}$$

(Suppl. Equation 4)

For a uniform distribution, the previous equation can be simplified to:

$$fraction\_uniform=1-\left( 1-p \right)^{k}$$

(Suppl. Equation 5)

**S3: Expected fraction of unique coupons is maximal when the collection is uniform and complete**

A collection that is both complete (it contains all coupons) and uniform (each coupon is sampled with the same probability) is the upper bound for any given $n>2$ and $k>1$. The demonstration follows:

First, we prove that a uniform distribution will have higher fraction than a non-uniform. Given a uniform distribution P with probability $p=1/n$ and a non-uniform distribution $Q=\{q_{1},\ldots,q_{n}\}$, both of size $n$ and $\sum_{i=1}^{n} q_{i}=1$, we want to prove the following inequality (from (Suppl. Equation 4) and (Suppl. Equation 5)):

$$1-\left( 1-p \right)^{k}=\frac{n-n\left( 1-p \right)^{k}}{n}\geq\frac{n- \sum_{i=1}^{n} {(1-q_{i})}^{k}}{n}$$

(Suppl. Equation 6)

Which is equivalent to:

$$\sum_{i=1}^{n} {(1-p)}^{k} \leq\sum_{i=1}^{n} {(1-q_{i})}^{k}$$

(Suppl. Equation 7)

We consider now that $\forall i<n-1; q_{i}=p$, we define $0<\varepsilon_{j}\leq p; q_{n-1}=p-\varepsilon$ and $q_{n}=p+\varepsilon$, making the distribution $Q$ quasi-uniform, except for the last two values. From this, we can write Suppl. (Suppl. Equation 7) as:

$$\left( \sum_{i=1}^{n-2} {(1-p)}^{k} \right)+\left( 1-p \right)^{k}+\left( 1-p \right)^{k}<\left( \sum_{i=1}^{n-2} \left( 1-p \right)^{k} \right)+\left( 1-p+\varepsilon\right)^{k}+\left( 1-p-\varepsilon\right)^{k}$$

(Suppl. Equation 8)

It can be easily seen that, due to the nature of the exponential function, and with $k>1$:

$$\left( 1-p \right)^{k}+\left( 1-p \right)^{k}<\left( 1-p+\varepsilon\right)^{k}+\left( 1-p-\varepsilon\right)^{k}$$

(Suppl. Equation 9)

Note that any non-uniform distribution can be built from a uniform distribution by adding as many $\varepsilon_{j}$ as necessary. Thus, we can conclude that any non-uniform distribution will have a lower fraction than the equivalent uniform distribution.

To show that non-complete distributions have lower fraction, we can see that it is simply a non-uniform distribution $Q$ with $1\leq i\leq n-l; q_{i}=\frac{1}{n-l}$ and $n-l<i\leq n; q_{i}=0$ and the same proof as before can be applied.
